# Low doses of ionizing radiation enhance angiogenesis and consequently accelerate post-embryonic development but not regeneration in zebrafish

**DOI:** 10.1038/s41598-020-60129-9

**Published:** 2020-02-21

**Authors:** Filipa G. Marques, Lara Carvalho, Joana S. Sousa, José Rino, Isabel Diegues, Esmeralda Poli, Filomena Pina, Leonor Saúde, Susana Constantino Rosa Santos

**Affiliations:** 1 Centro Cardiovascular da Universidade de Lisboa, Faculdade de Medicina, Universidade de Lisboa, Lisboa, Portugal. Av. Prof. Egas Moniz, 1649-028 Lisbon, Portugal; 2 Instituto de Medicina Molecular, Faculdade de Medicina, Universidade de Lisboa, Lisboa, Portugal. Av. Prof. Egas Moniz, 1649-028 Lisbon, Portugal; 3 Centro Hospitalar Universitário Lisboa Norte, Lisboa, Portugal. Av. Prof. Egas Moniz, 1649-035 Lisbon, Portugal

**Keywords:** Developmental biology, Angiogenesis

## Abstract

Low doses of ionizing radiation (LDIR) activate endothelial cells inducing angiogenesis. In zebrafish, LDIR induce vessel formation in the sub-intestinal vessels during post-embryonic development and enhance the inter-ray vessel density in adult fin regeneration. Since angiogenesis is a crucial process involved in both post-embryonic development and regeneration, herein we aimed to understand whether LDIR accelerate these physiological conditions. Our data show that LDIR upregulate the gene expression of several pro-angiogenic molecules, such as *flt1*, *kdr*, *angpt2a*, *tgfb2*, *fgf2* and *cyr61*in sorted endothelial cells from zebrafish larvae and this effect was abrogated by using a vascular endothelial growth factor receptor (VEGFR)-2 tyrosine kinase inhibitor. Irradiated zebrafish present normal indicators of developmental progress but, importantly LDIR accelerate post-embryonic development in a VEGFR-2 dependent signaling. Furthermore, our data show that LDIR do not accelerate regeneration after caudal fin amputation and the gene expression of the early stages markers of regeneration are not modulated by LDIR. Even though regeneration is considered as a recapitulation of embryonic development and LDIR induce angiogenesis in both conditions, our findings show that LDIR accelerate post-embryonic development but not regeneration. This highlights the importance of the physiological context for a specific phenotype promoted by LDIR.

## Introduction

Angiogenesis is the process by which new blood vessels form through pre-existing structures and is orchestrated by a fine tuned balance between pro- and anti-angiogenic factors^1^. By itself, this dynamic balance coordinates not only the behavior of endothelial cells, but also their interactions with the surrounding microenvironment^2^. Physiologically, angiogenesis plays a pivotal role during embryonic development, associated with organ growth, and in adulthood, during wound healing and the female reproductive system^3^. Furthermore, the regulatory protein imbalance may cause vascular regression, abnormal vessel remodeling, or excessive vessel growth, which are the mechanisms most associated with different angiogenesis-dependent diseases, such as tissue ischemia or cancer^4,5^. The molecular mechanisms underlying directly or indirectly the angiogenic process have been studied using several experimental models in which regulatory proteins associated with angiogenesis led to vessel abnormalities or lethality in early stages of development^6–9^. We have previously demonstrated that doses ionizing radiation lower that 0.8 Gy, defined as low doses of ionizing radiation (LDIR) induce angiogenesis^10,11^. The biological effects of high to moderate doses of ionizing radiation are different from those of moderate to low. Inflammation^12^, fibrosis^13^, senescence^14^ and vascular damage^15^ are induced by high to moderate doses of radiation. In contrast, low-dose radiation was described as anti-inflammatory^12^ and pro-angiogenic^10,11^. *In vitro*, we found that LDIR activate endothelial cells^11^ by inducing the phosphorylation of the vascular endothelial growth factor receptor (VEGFR), which consequently activates multiple signaling pathways and enhances a pro-angiogenic response. The endothelial gene expression profile showed a significant modulation by LDIR, where several receptors and proteins associated with a pro-angiogenic response were significantly up-regulated^10^. Consistently, in the presence of a VEGFR tyrosine kinase inhibitor, the effect observed upon LDIR exposure was abrogated, which was further validated using animal models^10,11^. In a hindlimb ischemia model, we demonstrated that angiogenesis and arteriogenesis was enhanced by LDIR and consequently neovascularization was induced^10^. In particular, using a zebrafish model we observed that LDIR induced vessel formation in the sub-intestinal vessels during post-embryonic development. These vessels were formed by sprouting angiogenesis, a process that was accelerated by LDIR without causing excessive vessel formation^11^. Moreover, we examined caudal fin regeneration in adult zebrafish and we observed that LDIR increased the inter-ray vessel density after regeneration in irradiated fins^11^. However, the cellular and molecular mechanisms associated with the biological effects of LDIR, during post-embryonic development and regeneration, are still poorly understood.

When dealing with ionizing radiation, it is usually assumed that there is no dose so low as not to have any effect (linear no-threshold hypothesis), assuming, as such, a linear radiation risk.

Recent experiments, however, have uncovered low-level effects, such as adaptative response^16–18^ or bystander effects^19–23^, that indicate that this hypothesis may not be valid when dealing with small dose exposure. As an example, there are low doses with stimulating effects and high doses with inhibitory ones, a phenomenon known as a biphasic dose response. In a recent study using zebrafish, the response was triphasic, showing an inhibition when doses are very low, a stimulation with low doses and an inhibition with high doses^24,25^.

This work shows that LDIR activate the endothelial cells and consequently accelerate embryonic development but not regeneration, even though LDIR significantly increased the density of vessels between caudal fin rays in the irradiated regenerated fin when compared to the non-irradiated one.

## Results

### LDIR significantly increase the expression of several pro-angiogenic targets in endothelial cells from zebrafish larvae in a VEGFR-dependent manner

**Figure 1:**
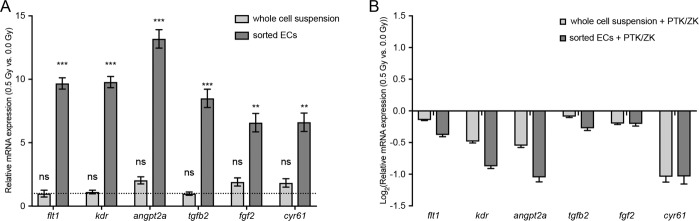
LDIR upregulate the expression of several pro-angiogenic factors in *Fli1:EGFP* zebrafish in a VEGFR-dependent manner. *Fli1:EGFP* zebrafish larvae were exposed or not to 0.5 Gy at 3, 4 and 5 dpf and pre-treated or not with PTK/ZK, 30 minutes before each irradiation. The mRNA expression of *flt1*, *kdr*, *angpt2a*, *tgfb2*, *fgf2*, and *cyr61* from whole cell suspension and endothelial sorter cells was quantified by qRT-PCR and normalized to *elongation factor 1*. (**A**) Data represent the relative mRNA levels of whole cell suspension and endothelial sorted cells from larvae exposed LDIR relative to the non-irradiated one (dashed line), in triplicate measurements. **P < 0.01; ***P < 0.001; ns, non-significant. (**B**) Data represent the fold change in mRNA expression of whole cell suspension and endothelial sorted cells of irradiated larvae exposed to PTK-ZK relative to non-irradiated and untreated ones, in triplicate measurements. Data are representative of six independent experiments.

We have previously showed that LDIR accelerate vessel formation by inducing angiogenic sprouting in zebrafish *Fli1*∶EGFP embryos^11^. In order to investigate if the endothelial gene expression was modulated by LDIR in the first 7 days of the embryonic development, the transgenic *Fli1:EGFP* zebrafish were used. At the 3^rd^ dpf, *Fli1:EGFP* zebrafish larvae were exposed or not to 0.5 Gy, during three consecutive days. At the 5^th^ dpf, the larvae were sacrificed. Gene expression was assessed for *flt1*, *kdr*, *angpt2a*, *tgfb2*, *fgf2*, and *cyr61* in a whole cell extract or endothelial sorted cells. A significant upregulation of *flt1*, *kdr*, *angpt2a*, *tgfb2*, *fgf2*, and *cyr61* were observed only when endothelial sorted cells were used (Fig. 1A). To test whether this effect was VEGFR-dependent, zebrafish larvae were treated λwith the VEGFR tyrosine kinase inhibitor (PTK/ZK) prior to each LDIR exposure. We found that the upregulation promoted by LDIR was completely abrogated by treatment with PTK/ZK (Fig. 1B).

Taken together, these data show that LDIR significantly increase the expression of several pro-angiogenic targets in endothelial cells from zebrafish larvae in a VEGFR-dependent manner.

### LDIR accelerate zebrafish post-embryonic development in a VEGFR-dependent manner

**Figure 2:**
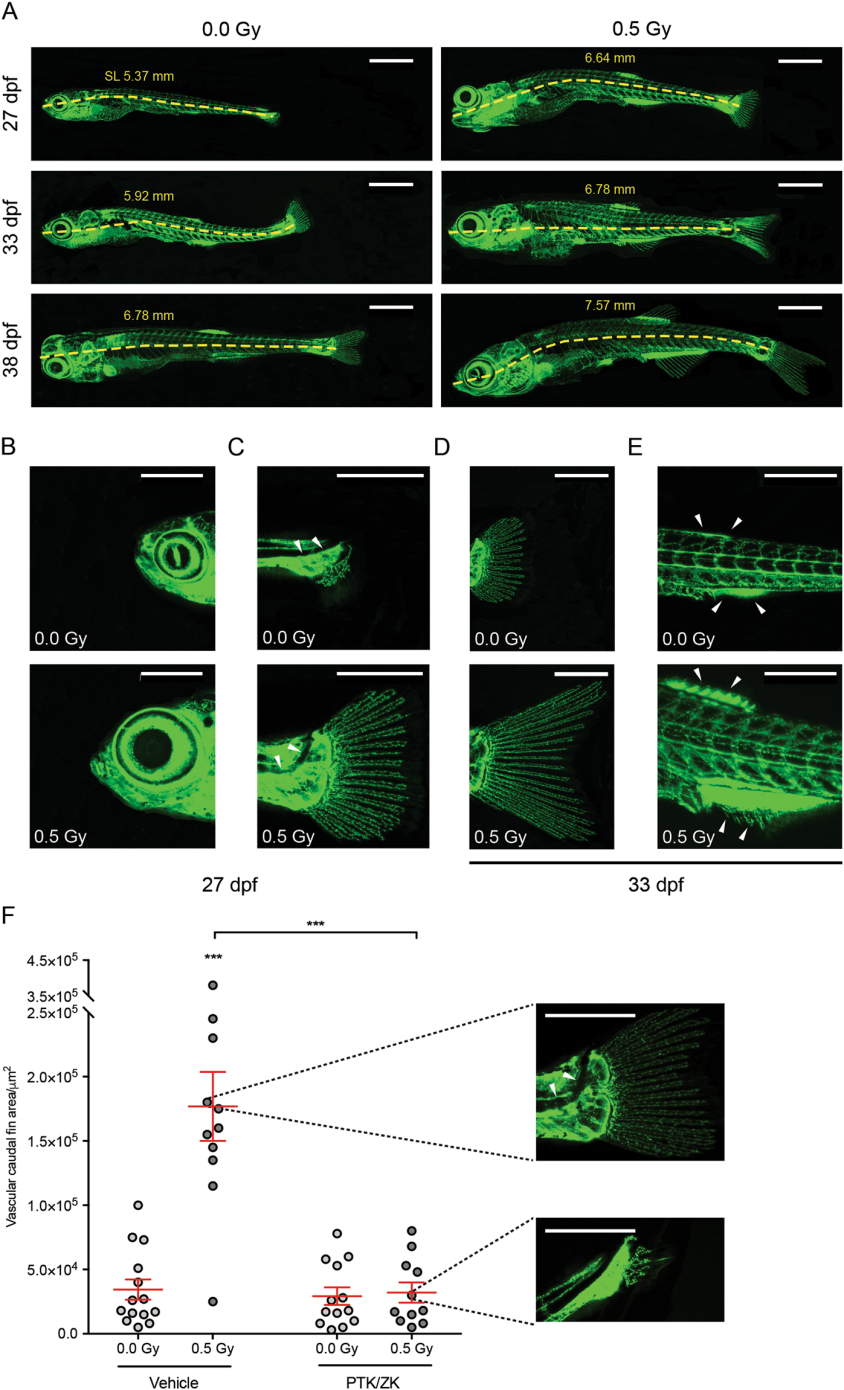
LDIR accelerate zebrafish development in a VEGFR-dependent manner. *Fli1:EGFP* zebrafish larvae were exposed or not to 0.5 Gy at 3, 4 and 5 dpf, pre-treated or not with PTK/ZK, 30 minutes before each irradiation and photographed over-time. (**A**) Representative images of the vasculature from non-irradiated and irradiated zebrafish at the 27^th^, 33^rd^ and 38^th^ dpf. The standard length (SL), in mm, was measured at each time-point for irradiated and non-irradiated zebrafish. (**B–D**) Post-embryonic development progress indicators were assessed at the 27^th^ and 33^rd^ dpf: (**B**) Head shape; (**C**) notochord flexion; (**D**) caudal fin; and (**E**) anal and dorsal fin. and Scale bars, 1 mm (**A**), 500 μm (**C,D**). (**F**) At the 33^rd^ dpf, developmental stage was established by quantification of vascular caudal fin area, using ImageJ. Representative images of the median phenotypes are showed next to the graph. Scale bars, 500 μm. Data are represented as mean ± SEM and two-way ANOVA test was used to determine differences between experimental groups; ***P < 0.001.

We previously found that LDIR activate endothelial cells and accelerate angiogenic sprouting in the first days post-fertilization^11^. Therefore, we assessed the effect of LDIR in the post-embryonic development. At the 3^rd^ dpf, *Fli1:EGFP* zebrafish larvae were exposed or not to 0.5 Gy, during three consecutive days and photographed over time. We found that irradiated zebrafish present an increase of their standard length (SL) at the 27^th^, 33^rd^ and 38^th^ dpf when compared to non-irradiated ones, suggesting that they reached a developmental milestone earlier in time (Fig. 2A). This finding was supported by other indicators of developmental progress such as head shape, notochord flexion and median fins (Fig. 2B–E). The irradiated zebrafish presented a more pronounced triangular head shape with a protruding anterior and a larger eye diameter (Fig. 2B) when compared to the non-irradiated zebrafish. The posterior notochord bends dorsally, a process termed flexion, which is more evident in irradiated zebrafish (flexion angle of ~ 45°) when compared to non-irradiated ones (Fig. 2C). Concerning the median fins, the irradiated caudal fin has a bilobate appearance with an intervening cleft suggesting that it achieved a later stage of development when compared to the non-irradiated caudal fin (Fig. 2D). The non-irradiated zebrafish present anal and dorsal fin condensation whereas the anal and dorsal fin rays are already evident in the irradiated zebrafish (Fig. 2E).

Taken together, our findings show that not only the irradiated zebrafish developed earlier in time as they present a normal developmental post-fertilization phenotype when compared to the non-irradiated zebrafish. Nevertheless, the differences observed between the 27^th^ and 38^th^ dpf between irradiated and non-irradiated experimental groups were negligible from 62 dpf on (data not shown). Moreover, we found that the acceleration of post-fertilization development promoted by LDIR was dependent of VEGFR, since PTK/ZK treatment prior to each LDIR exposure abrogated the effect promoted by LDIR, as illustrated and measured in Fig. 2F.

Since we showed that doses lower or equal to 0.8 Gy induce angiogenesis both *in vitro*^11^, in different animal models^10,11^ and in human^26^, we decided to consider this dose range and investigate the effect of doses lower or higher than 0.5 Gy in the zebrafish post-embryonic development. With the aim to investigate the effect of doses lower and higher than 0.5, *Fli1:EGFP* zebrafish larvae were exposed or not to 0.3 or 0.8 Gy, during three consecutive days and photographed over time. According to our results, Zebrafish exposed to 0.8 Gy present an increase of their SL at the 27^th^, 33^rd^ and 38^th^ dpf when compared to non-irradiated ones, results similar to those found in zebrafish exposed to 0.5 Gy (see Supplementary Fig. S1). However, at the same time points, the SL of Zebrafish exposed to 0.3 Gy were similar to non-irradiated zebrafish (see Supplementary Fig. S1). Consistently, we observed an up-regulation of *flt1*, *kdr*, *angpt2a*, *tgfb2*, *fgf2*, and *cyr61* in endothelial sorted cells from the zebrafish exposed with 0.8 Gy but not with 0.3 Gy (see Supplementary Fig. S1).

In conclusion our data show that zebrafish exposed to LDIR present similar vascular patterns when compared to non-irradiated ones but interestingly, the post-fertilization development is accelerated in the first 38 days post-fertilization by LDIR (0.5 Gy or 0.8 Gy) in a VEGFR-dependent manner.

### LDIR do not accelerate the adult caudal fin regeneration

**Figure 3:**
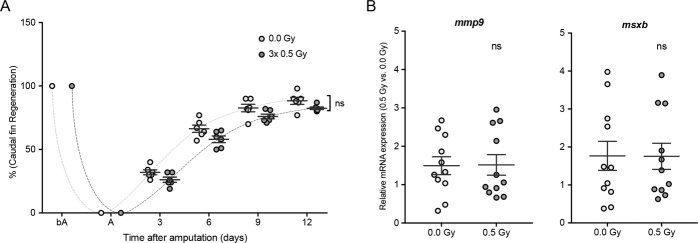
Wound healing and blastema formation, triggering the regeneration process, are not affected by LDIR after amputation in zebrafish. The caudal fin of adult *Fli1:EGFP* zebrafish was amputated at mid-fin level and immediately exposed or not to 0.5 Gy for 3 consecutive days. (**A**) Caudal fin regeneration was followed over time, and regenerated area was quantified (in pixels) using ImageJ software. Data are represented as mean ± SEM and two-way with repeated measures ANOVA test was used to determine differences between experimental groups; ns, non-significant. (**B**) The mRNA expression levels of *mmp9* (wound-healing marker) and *msxb* (blastema formation marker) were determined by qRT-PCR and normalized to *elongation factor 1*. Data represent the relative mRNA expression in irradiated tissue compared to non-irradiated ones. Data are represented as mean ± SEM of 12 independent zebrafish and differences between groups was assessed by independent samples T-test; ns, non-significant.

We have previously demonstrated a striking increase in the inter-ray vessel density in irradiated regenerated caudal fins upon LDIR exposure using the adult transgenic *Fli1:EGFP*^11^. In order to investigate whether LDIR could accelerate the caudal fin regeneration, the caudal fin was amputated at mid-fin level immediately before irradiation. During three consecutive days, the amputated caudal fin was exposed to 0.5 Gy and allowed to recover. The animals were followed over time and our data show that the recovery time to accomplish fin regeneration was not modulated by LDIR exposure (Fig. 3A). Moreover, we investigated if LDIR could modulate the early stages of regeneration by assessing the gene expression of markers associated with the initial wound healing (such as *mmp9*)^27^ and blastema formation (such as *msxb*)^28^. As shown in Fig. 3B, the gene expression of *mmp9*, a wound healing marker, was not affected by LDIR exposure at 8 hours post-amputation. Furthermore, LDIR did not modulate the expression of *msxb*, a blastema formation marker, at 24 hours post-amputation. Taken together, our data suggest that LDIR do not affect the early stages of regeneration and do not accelerate regeneration.

## Discussion

Low doses of ionizing radiation (LDIR), inferior to 0.8 Gy, induce a fast phosphorylation of multiple endothelial proteins such as VEGFR-2, that plays a major role in angiogenesis^11^. In consequence, the activation of PI3K/AKT and ERK/MAPK signaling pathways was observed and the expression of 2374 genes was modulated by LDIR^11^ in human microvasculature. Interestingly, 1344 of those genes, including angiogenic ones, have their expression significantly increased after LDIR exposure^10^.

Moreover, in hypoxic mimicking conditions LDIR induce VEGF production^11^. By activating the VEGFR-2, LDIR enhance endothelial cell migration *in vitro*^11^. *In vivo*, LDIR significantly promote adult angiogenesis in the murine Matrigel assay and improved blood circulation, increased microvasculature and arteriogenesis in a hindlimb ischemia experimental model^10,11^. In human, we validated that endothelial cells were activated and vessel density increased by LDIR in biopsies from tissues exposed to these LDIR^26^.

In zebrafish, we found that LDIR accelerate angiogenic sprouting and promote adult angiogenesis during embryonic development and fin regeneration, respectively^11^. In the literature it is accepted that there are significant parallels between how tissues are built in embryonic morphogenesis and how they are rebuilt during tissue regeneration after wound healing^29^. Since angiogenesis is a crucial process involved in both conditions, herein we aimed to understand if LDIR accelerate embryonic development by enhancing angiogenesis sprouting in the initial stages of development and to evaluate if the caudal fin regeneration could also be accelerated by LDIR.

Consistent with our previous results showing that LDIR potentiate an angiogenic response, where multiple signaling pathways and pro-angiogenic targets are induced in endothelial cells both *in vitro* and *in vivo*^10,11^, herein we observed an upregulation of *flt1*, *kdr*, *angpt2a*, *tgfb2*, *fgf2*, and *cyr61* in endothelial cells from the transgenic zebrafish line *Fli1*:EGFP upon 0.5 Gy exposure over three consecutive days. Our data do not exclude the possibility that other cells in addition to endothelial ones, could be modulated by LDIR contributing for a pro-angiogenic response, but strongly suggest the involvement of endothelial cells. It was described that ionizing radiation modulate inflammation acting in the immune cells^30^. However, this response is dependent on the dose and while doses lower than 1 Gy are anti-inflammatory^30^, doses higher than 2 Gy are pro-inflammatory^31^. The efficiency of LDIR was already described in mice models of inducible arthritis^32^. In a context of hindlimb ischemia, we described that CD45+ cell infiltrate was significantly recruited to the injured muscle, a process that is significantly inhibited upon LDIR exposure^10^. It is important to note that the tissue homeostasis will be determinant for the effect of LDIR. LDIR act synergistically with hindlimb ischemia, inducing a pro-angiogenic phenotype. In contrast, LDIR do not affect the non-ischemic vasculature^10^. In zebrafish, LDIR significantly increased the inter-ray density in the regenerated caudal fin but have no effect in non-amputated caudal fin^11^. Consistently with our previously data demonstrating that LDIR induced angiogenic sprouting in the first days of the embryonic development^11^, herein we show that LDIR significantly increase the expression of pro-angiogenic genes in endothelial cells in a VEGFR dependent signaling cascade, since PTK/ZK abrogated the LDIR effect. Consistently, we also show that LDIR accelerate the post-embryonic development in the first 38 days post-fertilization, the difference becoming less notorious on the following days and no longer detected on day 64. Zebrafish exhibit a normal ageing process (data not shown) and the analysis of the different indicators of developmental progress such as head shape, notochord flexion and median fins show that irradiated zebrafish present a normal post-embryonic development. Furthermore, it is interesting to note that PTK/ZK abrogate the effect on post-embryonic acceleration induced by LDIR. This effect is not dependent of PTK/ZK *per se*, since untreated and PTK/ZK treated zebrafish larvae present similar phenotype as assessed by vascular caudal fin area quantification.

In this work our data also show that fin regeneration was not accelerated by LDIR and markers of the early stages of regeneration (wound healing and blastema formation) were not modulated by LDIR. Accordingly, Bayliss *et al*. showed that endothelial cells are not required for the initial stages of zebrafish fin regeneration and PTK/ZK does not affect genes required for regeneration such as *msxb*^33^. However, even if a small amount of avascular tissue, up to ~1 mm, can be regenerated in the absence of blood vessels growth, angiogenesis is crucial to regenerate beyond this size limit^33^. Consistently, our previous data show that LDIR significantly increase the inter-ray vessel density in regenerated caudal fin when compared to non-irradiated regenerated ones^11^. This effect could be due to a synergistic effect of LDIR and hypoxia immediately after amputation, since it was found that *vegf-a* is induced by LDIR under hypoxic conditions^11^ and it is expressed in early stages of regeneration in contrast to *vegfr2*, not detected until 2 days after amputation^33^.

Taken together, our data show that LDIR act both in post-embryonic development and fin regeneration, since both processes are dependent of angiogenesis. However, it is interesting to note that LDIR as a pro-angiogenic stimuli accelerate post-embryonic development but not fin regeneration even if repair is often considered a recapitulation of morphogenesis. This suggests that although LDIR induce angiogenesis, their biological consequence could be dependent of the physiological context.

## Material and Methods

### Ethics statement

All animal procedures were performed according to Directive 2010/63/ EU. The procedures were approved by the institutional Animal Welfare Body, licensed by DGAV, the Portuguese competent authority for animal protection (license number 023861/2013).

### Zebrafish line and maintenance

Transgenic *Fli1:EGFP* zebrafish were obtained from the Zebrafish International Resource Centre and maintained under standard conditions in the institutional animal care facility. Zebrafish larvae were irradiated at the 3^rd^ day post-fertilization (dpf). The caudal fin amputation was performed in 10-month-old adult zebrafish. During experiments, animals were maintained in separate tanks (two adult individuals per tank or twenty larvae per tank). Zebrafish larvae and adults were anaesthetized with Tricaine 1×(Sigma-Aldrich), before irradiation, amputation or imaging.

### Zebrafish caudal fin amputation and regenerated area quantification

The adult zebrafish caudal fin was amputated three segments below the most proximal bony ray bifurcation, using the guillotine method with a razor blade immediately before irradiation and allowed to recover at 33 °C after LDIR exposure. Phase-contrast full-fin images were taken before amputation, immediately after amputation and every 3 days until the caudal fin was fully regenerated at day 16 post-amputation. Before amputation, the caudal fin full area (in pixels) from the proximal end of the fin rays to the distal fin edge was quantified for each animal, using Image J software (NIH). The regenerated area, from the amputation plane to the distal tip, was also quantified for each animal at every time-point. Since zebrafish are very heterogeneous regarding its size, every regenerated area measurement was corrected to the uncut fin size.

### Drug administration

Zebrafish larvae were subjected to PTK787/ZK222584 (PTK/ZK; Novartis) treatment or its vehicle (polyethylene glycol-300 from Sigma) 30 minutes before each irradiation, which was added to embryo medium, and then transferred to embryo medium.

### Irradiation

Anaesthetized zebrafish larvae and adults were transferred to an acrylic phantom box to achieve a certain thickness and exposed to LDIR as described previously^11^. Briefly, a computed tomography (CT) scan (Somatom Sensation, Siemens) was performed and a volumetric acquisition was carried out; acquired images were reconstructed with axial slices width of 1 mm, and cross sectional data was transferred to the image processing system work station for contouring the planning target volume (PTV). The radiotherapy plan was devised on a dedicated 3D planning system (PLATO, Nucletron) using an isocentric dose distribution of two opposite fields (0°, 180°) at 6 MV energy, normalized to a reference point. IR delivery was performed at room temperature using a linear accelerator x-rays photon beam (Varian Clinac 2100 CD) operating at a dose rate of 300 MU/min. A 0.6 cm^3^ PTW farmer ionizing chamber, connected to UNIDOS electrometers, was used to validate the IR doses calculated by PLATO, according to the IAEA TRS-398 protocol. We obtained, in average, differences lower than 2% between the experimental and the PLATO planning system dose values.

### Zebrafish imaging

Images from zebrafish larvae were acquired on a Zeiss LSM 510 META using a 20×/0.80 objective and the 488 nm laser line of an Ar laser in conjunction with a LP 505 nm filter. Caudal fin images were acquired on a brightfield Olympus MVX10 MacroZoom microscope using a MV PLAPO 1x objective. All zebrafish experiments were followed over time.

### Cell suspension and endothelial cells isolation by fluorescence-activated cell sorting (FACS)

Wild type *TG(fli1:EGFP)*^*y1*^ larvae were euthanized using a lethal concentration of Tricaine (25×; Sigma-Aldrich) at the 5^th^ dpf and treated with 0.5% trypsin-EDTA (Invitrogen) solution at 37 °C for 45 minutes for tissue disaggregation. Cell suspensions were then filtered using 70 μm cell strainer (BD Falcon™), washed twice and resuspended in PBS containing 5% FBS. GFP-positive endothelial cells were isolated and collected using a FACSAriaIII (Becton Dickinson) with a blue laser with 488 nm and 20 mW power. FACS was performed at room temperature under sterilized conditions. GFP+ cells were collected in PBS containing 5%FBS. Following sorter, cell viability was greater than 90% and 5 × 10^5^ GFP+ from approximately 300 *Tg(fli1:egfp)*^*y1*^ embryos. The total mRNA from zebrafish endothelial sorted cells and zebrafish whole larvae cell suspension was extracted using the RNeasy® Micro Kit (QIAGEN), following instructions provided by the manufacturer, including addition of an on-column DNase-I treatment. cDNA was synthesized using the RT^2^ Nano PreAmpTM cDNA synthesis Kit (SABiosciences, QIAGEN) according to manufacturer’s protocol.

### Regenerated areas extraction

The regenerated areas corresponding to the wound-healed and blastema portions of the caudal fin were cut 8 and 24 hours, respectively, after amputation and placed immediately in Trizol (Invitrogen). Total mRNA isolation from regenerated areas was performed using the Total isolation Trizol reagent protocol (Invitrogen). Briefly, after samples in Trizol were thawed on ice, 200 μL of chloroform/mL of Trizol was added followed by a vigorous agitation and a 5-minute incubation at room temperature. The aqueous phase was precipitated with 0.5 mL of isopropanol for 10 minutes at room temperature between two 10-minute centrifugations, at 13000 rpm and 4 °C. The RNA pellet was washed with 75% ethanol and centrifuged for 10 minutes, at 13000 rpm and 4 °C. The dried RNA pellet was resuspended in 20–30 μL of RNAse-free DEPC (diethylpyrocarbonate)-treated H_2_O and incubated at 65 °C for 10 minutes. Samples purity was evaluated based on A260/A280 ratio. cDNA was synthesized, after DNase treatment with DNA-*free*™ DNA Removal Kit (Ambion, Invitrogen) according to manufacturer’s protocol, using a SuperScriptTM II Rnase H-Reverse Transcriptase Kit (Invitrogen).

### Quantitative real-time PCR

The mRNA expression levels of all targets were assessed by quantitative Real-Time PCR (qRT-PCR), which was performed using the Power SYBR® Green system (Invitrogen) on a 7500/7500 Fast Real-Time PCR System (Applied Biosystems). Gene-specific primer pairs are presented as follows (5′ – 3′):

*flt1* Fw: GTC ACT AAC CCA GAC GCC AAA G;

*flt1* Rev: ATG AAT CCC TGC CTG CTG TT;

*kdr* Fw: TCT TCA CTC TTC ACG TGC TTT TTA G;

*kdr* Rev: GAA GGT GTG TAT CTC CAT CAG GAA;

*angpt2a* Fw: ACT GAC AGA TGT GGA GAC GCA;

*angpt2a* Rev: GTG CTC AGA GAG TAC TCC AGC;

*tgfb2* Fw: TGA CGT CAC CTA CAC AGC G;

*tgfb2* Rev: ACA GTG CAG GCT GAT CTT GA;

*fgf2* Fw: ACG CAG ACG GAC GAC TGT T;

*fgf2* Rev: GGA TAC TTG CGG GAT CTG TAT G;

*cyr61* Fw: GCG GAG ACT CGG AGA AAG AAC;

*cyr61* Rev: CGA TGC ACT TCT CCA TCT GAT;

*mmp9* Fw: CTG GGC ACC TGC TCG TTG;

*mmp9* Rev: ATT GGA GAT GAC CGC CTG C;

*msxb* Fw: AGG AAC AGA GCA CTT GGT CAA ACT;

*msxb* Rev: TGA GGT TGA GGG AGT TGG AGA AC;

*elong1* Fw: ACG CCC TCC TGG CTT TCA CCC;

*elong1* Rev: TGG GAC GAA GGC AAC ACT GGC.

The thermal profile run of the method consisted of one holding stage of 2 minutes, at 50 °C and 10 minutes, at 95 °C, followed by 50 cycles of 15 seconds, at 95 °C and 30 seconds, at 60 °C. Further analysis was performed using RT-PCR 7500 Fast software (Applied Biosystems) and *elong1* was used as a housekeeping gene to normalize the quantification. Note that the levels of *elong1* were not modulated by irradiation *per se*. The relative quantification was performed according to the comparative 2^−ΔΔC^^T^ method^34,35^.

### Statistical analysis

For statistical analysis, data were analyzed using SPPS software (v.20). Normality was determined for all numeric data by Shapiro-Wilk test. As a normal distribution could be assumed, differences between experimental groups were identified with Student’s t-test for independent samples, while Mann-Whitney U test was used as normality could not be assumed. The differences between and within subjects’ regarding the caudal fin vascular area were determined by using a Two-way ANOVA test. Two-way repeated measures ANOVA was used to clarify differences between groups before and after caudal fin amputation and regeneration. *p-values* lower than 0.05 were considered statistically significant.

## Supplementary information

Supplementary information.
