# Supplementary material for: Low doses of ionizing radiation enhance angiogenesis and consequently accelerate post-embryonic development but not regeneration in zebrafish
Source: Sci Rep. 2020 Feb 21;10:3137. doi: 10.1038/s41598-020-60129-9 (PMC7035379; doi:10.1038/s41598-020-60129-9)
Supplement: Supplementary file 1 — Supplementary information. [file 41598_2020_60129_MOESM1_ESM.pdf]

Supplementary information

Low doses of ionizing radiation enhance angiogenesis and consequently accelerate post-embryonic development but not regeneration in zebrafish

Filipa G. Marques[1], Lara Carvalho[2†], Joana S. Sousa[1†], José Rino[2], Isabel Diegues[3], Esmeralda Poli[3], Filomena Pina[3], Leonor Saúde[2], Susana Constantino Rosa Santos[1*]

[1]Centro Cardiovascular da Universidade de Lisboa, Faculdade de Medicina, Universidade de Lisboa, Lisboa, Portugal. Av. Prof. Egas Moniz, 1649-028 Lisbon, Portugal.

[2]Instituto de Medicina Molecular, Faculdade de Medicina, Universidade de Lisboa, Lisboa, Portugal. Av. Prof. Egas Moniz, 1649-028 Lisbon, Portugal.

[3]Centro Hospitalar Universitário Lisboa Norte, Lisboa, Portugal. Av. Prof. Egas Moniz, 1649-035 Lisbon, Portugal.

†The second and third authors contributed equally to the study

Filipa G. Marques: filipa.gil.marques@gmail.com

Lara Carvalho: laramcarv@gmail.com

Joana S. Sousa: josousa@biophys.mpg.de

Isabel Diegues: isabel.diegues@chln.min-saude.pt

Filomena Pina: filomena.pina@chln.min-saude.pt

Esmeralda Poli: esmeralda.poli@chln.min-saude.pt

José Rino: Joserino@medicina.ulisboa.pt

Leonor Saúde: msaude@medicina.ulisboa.pt

* Corresponding author

Address Correspondence to: Susana Constantino Rosa Santos; sconstantino@medicina.ulisboa.pt

Faculdade de Medicina da Universidade de Lisboa. Avenida Prof. Egas Moniz. Edificio Egas Moniz 1649-028 Lisboa. Fax: +351 217 999 477; Tel: +351 217 999 481

This PDF file includes:

Supplementary Figure 1

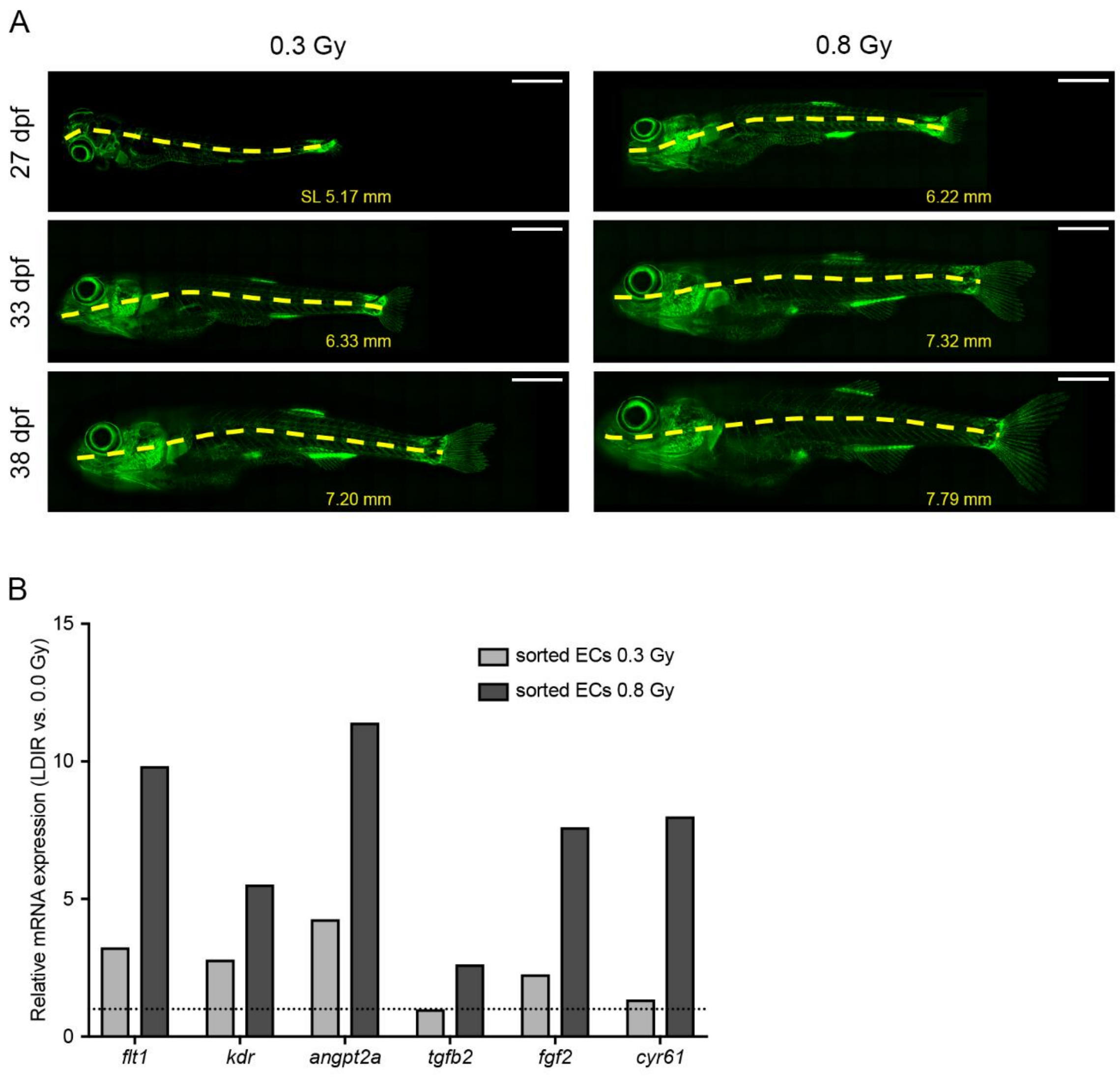

Figure S1 – Rates of LDIR in the zebrafish development *Fli1:EGFP* zebrafish larvae were exposed or not to 0.3 or 0.8 Gy at 3, 4 and 5 dpf and photographed over-time. (A) Representative images of the vasculature from zebrafish exposed to 0.3 or 0.8 Gy at the 27th, 33rd and 38th dpf. The standard length (SL), in mm, was measured at each time-point for both irradiated experimental conditions. (B) The mRNA expression of *flt1*, *kdr*, *angpt2a*, *tgfb2*, *fgf2*, and *cyr61* from endothelial sorter cells was quantified by qRT-PCR and normalized to *elongation factor 1*. Data represent the relative mRNA levels of endothelial sorted cells from larvae exposed to 0.3 or 0.8 Gy relative to the non-irradiated one (dashed line).
